# On the Blood in Bodies Killed by Strangling

**Published:** 1846-12

**Authors:** 


*On the Blood in bodies killed by Strangling.*—Ciccone made the following communication to the scientific assocoation at Naples. After he had observed that in persons destroyed by asphyxia, the quantity of fibrine in the blood is diminished, and that the circulation continues for some time after respiration has become suspended, he farther found that in cases where the respiration, as also the return of the blood in the jugular veins to the heart, had become prevented by a ligature placed round the neck, whilst the greater circulation, and hence also the metamorphosis of arterial into venous blood, which, although now imperfect, takes place in the capillary vessels, the blood accumulating in the jugular veins, that portion of this fluid which is above the ligature contains the normal quantity of fibrine, while in that which is below the ligature the quantity is diminished. This observation is thus far of value, that if the body has not been killed by strangling, and the ligature has not been applied until after death, these changes in the blood will not be found. At the same meeting Cappa detailed his experiments; he found that in ten strangled fowls, death was in six instances caused by asphyxia and apoplexy combined; twice by asphyxia alone; and twice by apoplexy alone. In the last cases, on the microscopic examination of the blood above the ligature, its corpuscles were found distended, and generally without a central nucleus; some of them were elongated, and others again in conglomerated masses, while the blood corpuscles in all the other parts of the body presented their natural appearance. Where death had been the result of asphyxia alone, all these alterations in the corpuscles were, on the contrary, observed only in the parts of the body below the ligature; and lastly, where death had been caused by a complication of apoplexy and asphyxia, the alterations in the blood corpuscles could be seen in all parts of the body without distinction. The above test could therefore, in his opinion, be applicable only in the two first circumstances, viz: where death was from apoplexy or asphyxia alone, and not where it was caused by these two conjoined, which occurrence was by far the most frequent. Where death was the result of apoplexy, he had certainly seen the jugular veins thicker and more full of fibrin, and he had seen exactly the reverse where asphyxia had been the cause; but where death was accompanied with symptoms of apoplexy and asphyxia conjoined, this test was of no avail.—*Monthly Jour. Med. Science*, from *Annali Univ. de Med.—Ibid.*

